# Leveraging gains from African Center for Integrated Laboratory Training to combat HIV epidemic in sub-Saharan Africa

**DOI:** 10.1186/s12913-020-06005-8

**Published:** 2021-01-06

**Authors:** Ritu Shrivastava, Richard Poxon, Erin Rottinghaus, Leyya Essop, Victoria Sanon, Zawadi Chipeta, Elsie van-Schalkwyk, Phuti Sekwadi, Pelagia Murangandi, Shon Nguyen, Josh Devos, Shanna Nesby-Odell, Thomas Stevens, Farouk Umaru, Alex Cox, Andrea Kim, Chunfu Yang, Linda M. Parsons, Babatyi Malope-Kgokong, John N. Nkengasong

**Affiliations:** 1 International Laboratory Branch (ILB), Division of Global HIV and TB (DGHT), Centres for Disease Control and Prevention (CDC), 1600 Clifton Road NE, Atlanta, GA 30333 USA; 2 African Center for Integrated Laboratory Training at National Health Laboratory Service, Johannesburg, South Africa; 3 Georgia State University, intern at ILB, DGHT, CDC, Atlanta, USA; 4 Laboratory Branch, DGHT, CDC, Pretoria, South Africa; 5 Supply Chain Management System, United States Agency for Internationl Development, Atlanta, USA; 6 DGHT, CDC, Atlanta, USA

**Keywords:** Laboratory system strengthening, Health worker training, Training assessment, Training effectiveness, Kirkpatrick model, Quality of testing, Leverage

## Abstract

**Background:**

In sub-Saharan Africa, there is dearth of trained laboratorians and strengthened laboratory systems to provide adequate and quality laboratory services for enhanced HIV control. In response to this challenge, in 2007, the African Centre for Integrated Laboratory Training (ACILT) was established in South Africa with a mission to train staffs from countries with high burdens of diseases in skills needed to strengthen sustainable laboratory systems. This study was undertaken to assess the transference of newly gained knowledge and skills to other laboratory staff, and to identify enabling and obstructive factors to their implementation.

**Methods:**

We used Kirkpatrick model to determine training effectiveness by assessing the transference of newly gained knowledge and skills to participant’s work environment, along with measuring enabling and obstructive factors. In addition to regular course evaluations at ACILT (pre and post training), in 2015 we sent e-questionnaires to 867 participants in 43 countries for course participation between 2008 and 2014. Diagnostics courses included Viral Load, and systems strengthening included strategic planning and Biosafety and Biosecurity. SAS v9.44 and Excel were used to analyze retrospective de-identified data collected at six months pre and post-training.

**Results:**

Of the 867 participants, 203 (23.4%) responded and reported average improvements in accuracy and timeliness in Viral Load programs and to systems strengthening. For Viral Load testing, frequency of corrective action for unsatisfactory proficiency scores improved from 57 to 91%, testing error rates reduced from 12.9% to 4.9%; 88% responders contributed to the first national strategic plan development and 91% developed strategies to mitigate biosafety risks in their institutions. Key enabling factors were team and management support, and key obstructive factors included insufficient resources and staff’s resistance to change.

**Conclusions:**

Training at ACILT had a documented positive impact on strengthening the laboratory capacity and laboratory workforce and substantial cost savings. ACILT’s investment produced a multiplier effect whereby national laboratory systems, personnel and leadership reaped training benefits. This laboratory training centre with a global clientele contributed to improve existing laboratory services, systems and networks for the HIV epidemic and is now being leveraged for COVID-19 testing that has infected 41,332,899 people globally.

**Supplementary Information:**

The online version contains supplementary material available at 10.1186/s12913-020-06005-8.

## Background

The role of public health laboratories is unparalleled in the global detection, prevention and treatment of communicable diseases. However, laboratory service delivery remains substandard worldwide, predominantly in low and middle-income countries [1]. Dearth of trained and adequate laboratory workforce has hampered success of many programs for years including Human Immunodeficiency Virus (HIV) and tuberculosis (TB) [2]. The need for accessible quality laboratory services is critical in the fight against the HIV, TB and malaria, and for the curtailment of morbidity and mortality to achieve their epidemic control [3–5].

In sub-Saharan Africa (sSA) where many countries face high burdens of infectious diseases, three main issues have plagued the laboratory response to the HIV, TB and malaria epidemics: 1) lack of financial support for laboratory systems, 2) lack of emphasis on quality laboratory policies and procedures, and 3) lack of adequate numbers of trained and certified laboratory personnel [6–8]. The consequences of these are extended turnaround times, laboratory testing errors, lack of support for functional continual quality improvement (CQI) and accreditation efforts [6, 7]. All of these shortcomings have resulted in a lack of confidence in laboratory services by physicians, other health care providers and the patients themselves [7, 8].

Over the past decade, there have been substantial investments in HIV prevention and treatment programs in high HIV burden countries [9, 10]. Despite these investments and technical support, shortages are still evident in the numbers of qualified laboratorians and management personnel, adequate laboratory infrastructures, and national policies and regulations that govern the quality of laboratory services [10–17].

In 2007, the U.S. Centers for Disease Control and Prevention (CDC), the President’s Emergency Plan for AIDS Relief (PEPFAR), and the South African National Health Laboratory Service launched the African Centre for Integrated Laboratory Training (ACILT) in Johannesburg, South Africa. The objective of ACILT was to develop competent laboratory workforces and strengthen laboratory systems through free, hands-on training courses for laboratory technical and national administrative personnel from sSA countries to accurately detect and monitor patients with HIV, TB and malarial infections. The participants were expected to integrate, and transfer knowledge gained to their home laboratory and administrative staffs and to collaborate with their Ministry of Health (MoH) to build country ownership.

Following nine years of ACILT’s operations (2007–2016), we sought to conduct a comprehensive evaluation to determine the impact in improving laboratory services by transferring knowledge and skills gained at ACILT by the trainees to strengthen in-country laboratories and national laboratory systems, and ultimately leading to improved patient services. To assess the effectiveness of the training courses, we designed and implemented a set of structured questionnaires to answer the question: “*Are PEPFAR and other partners’ investments in ACILT having a positive impact in building country ownership to provide quality and timely laboratory services to patients in countries with high burdens of infectious disease?”*

## Methods

### Study design

A retrospective and cross-sectional study was designed using levels 3 and 4 of the Kirkpatrick models [18]. Kirkpatrick model is the most recognized method of evaluating the effectiveness of training programs. The four levels of evaluation are: (1) the reaction of the student and their thoughts about the training experience; (2) the student’s resulting learning and increase in knowledge from the training experience; (3) the student’s behavioral change and improvement after applying the skills on the job and (4) the results or effects that student’s performance has on the business/organization. Levels 1 and 2 of the Kirkpatrick models were evaluated before and are not part of this study.

### Evaluation period

In 2015, structured questionnaires were sent for courses offered between 2008 and 2014.

### Training effectiveness

In this study training effectiveness was defined as the extent to which course participants use their newly gained knowledge, skills and behaviors in their workplace. In this study behaviors imply that the trainees successfully transferred the acquired knowledge, skills to others in their organizations which translated into patient service improvements.

### Study population

In 2015, questionnaires were sent to all 867 participants from 43 countries who attended 75 of the courses offered between 2008 and 2014. 203/867 responded making them the population for this study. Participants included laboratory professionals working in national reference laboratories, hospital laboratories, health centers, and faith-based organizations as well as public health professionals working in advisory and management capacities as consultants to MoH on national laboratory systems and networks.

### Included courses

Established in 2007 and operated through 2016, ACILT trained 2052 participants from 54 countries in 162 course offerings for 17 subject areas. We included all 867 participants in the evaluation who attended 75/162 course offerings in seven subject areas. Three of the seven courses were in the laboratory diagnostics category - HIV Viral Load/Early Infant Diagnosis (VL/EID), HIV Drug Resistance and HIV Incidence Assay. Four courses were in the system strengthening category, and included National Laboratory Strategic Planning, Laboratory Information Systems, Supply Chain Management Systems and Biosafety and Biosecurity. Courses related to TB, malaria, HIV Limiting-Antigen Avidity Assay, Grant & Proposal Writing, Bio-Risk Management were not included in this study since evaluations were either reported previously (Strengthening Laboratory Management Toward Accreditation - SLMTA) [19] or will be reported separately in the future.

### Development of questionnaires

Course Questionnaires (also attached as supplementary material) were developed in English with input from Monitoring and Evaluation advisors, course instructors and subject matter experts from the International Laboratory Branch (ILB) in the Division of Global HIV and TB at CDC Atlanta, the Association of Public Health Laboratories, the Partnership for Supply Chain Management Systems and the United States Agency for International Development (USAID). All questionnaires shared a common framework and were structured into sections for Demographics, Transfer of Applied Skills and Knowledge, Change in Results, Success and Challenges and Recommendations. With the exception of success and challenges to transfer knowledge and the recommendation section to improve course in the future, none had open-ended questions. These seven unique but similarly structured questionnaires had specific questions customized for each of the subject matter areas. The questionnaires were piloted with laboratory professionals who did not participate in the training.

### Data collection

In 2015 after obtaining voluntary consents, online Survey Gizmo (https://forms.surveygizmo.com/plans-pricing/) or paper-based survey questionnaires were sent to the participants with a two-week deadline for response. Study co-ordinators sent two follow-up reminders at 14 and 21 days to non-responders. For those participants with poor internet connections, 35 resident CDC Laboratory advisors were contacted to deliver questionnaires to consenting participants and then securely e-mailed the response to the study co-ordinators who entered into the database. Survey Gizmo allowed each registered responder to submit one response, ensuring each participant only responded once. Access to Survey Gizmo was password protected.

### Data analysis

Survey Gizmo collated quantitative data and collected up to 250 words for qualitative data in real-time. We measured the training effectiveness by the change in percentage (absolute change) in CQI indicators collected six months before (baseline) the training at ACILT and after the training. ACILT’s team shared de-identified data with CDC headquarter and CDC South Africa study team for analysis. The analyses were conducted with SAS v9.44 and Microsoft Excel.

### Qualitative data

With an aim to minimize bias in the analysis, the compiled data were examined independently by two study teams and categories or themes were created to bring several initial codes together. Based on team’s knowledge and experience these categories were then divided into sub-categories. Cumulative responses identified the most relevant categories. Following this approach we captured key positive factors and challenges affecting the transfer of knowledge in the field.

### Quantitative data

A participant was counted as a responder if they returned the completed survey. The analysis was performed on individual questions and was limited to the responders that completed the particular question. To aggregate the responses for a question in Microsoft Excel, the sum of affirmative responses was used as the numerator and the sum of responders who attempted the question was used as the denominator. “Not applicable” response to a question was included as a valid response. We analyzed responses by training course categories and subdivided them into laboratory diagnostic and system strengthening courses.

The laboratory diagnostic category courses included responses from VL/EID, HIV Drug Resistance and HIV Incidence Assays and captured CQI indicators for a number of standard operating procedures added or modified, rarely (25%) repeating assays due to poor or failed results, taking corrective action following proficiency scores less than 80% in participant’s own laboratories, average turnaround time (days) to report results. Turnaround time was measured from time of collection of specimens to return of results. We also captured change in error rates for VL/EID testing by causes, before and after the training.

The system strengthening responses were analyzed into two groups. The first group included courses for National Laboratory Strategic Planning, Laboratory Information Systems, and Supply Chain Management Systems. Among the post-course indicators we captured number of participants contributing to development of national plans and those with access to > 25% of needed resources for strategic planning. The second group consisted of responses for Biosafety and Biosecurity program by measuring improvements in participant’s facility using change in percentage (absolute change) before and after course offered at ACILT.

Since the responses were self-reported we attempted to corroborate the data by sharing them with 35 subject matter experts at ILB, CDC Atlanta, who have been serving as technical experts for PEPFAR-supported countries. They make frequent visits with in-country laboratory-support team and use standardized tools [20–22] to measure progress on CQI.

## Results

**Fig. 1:**
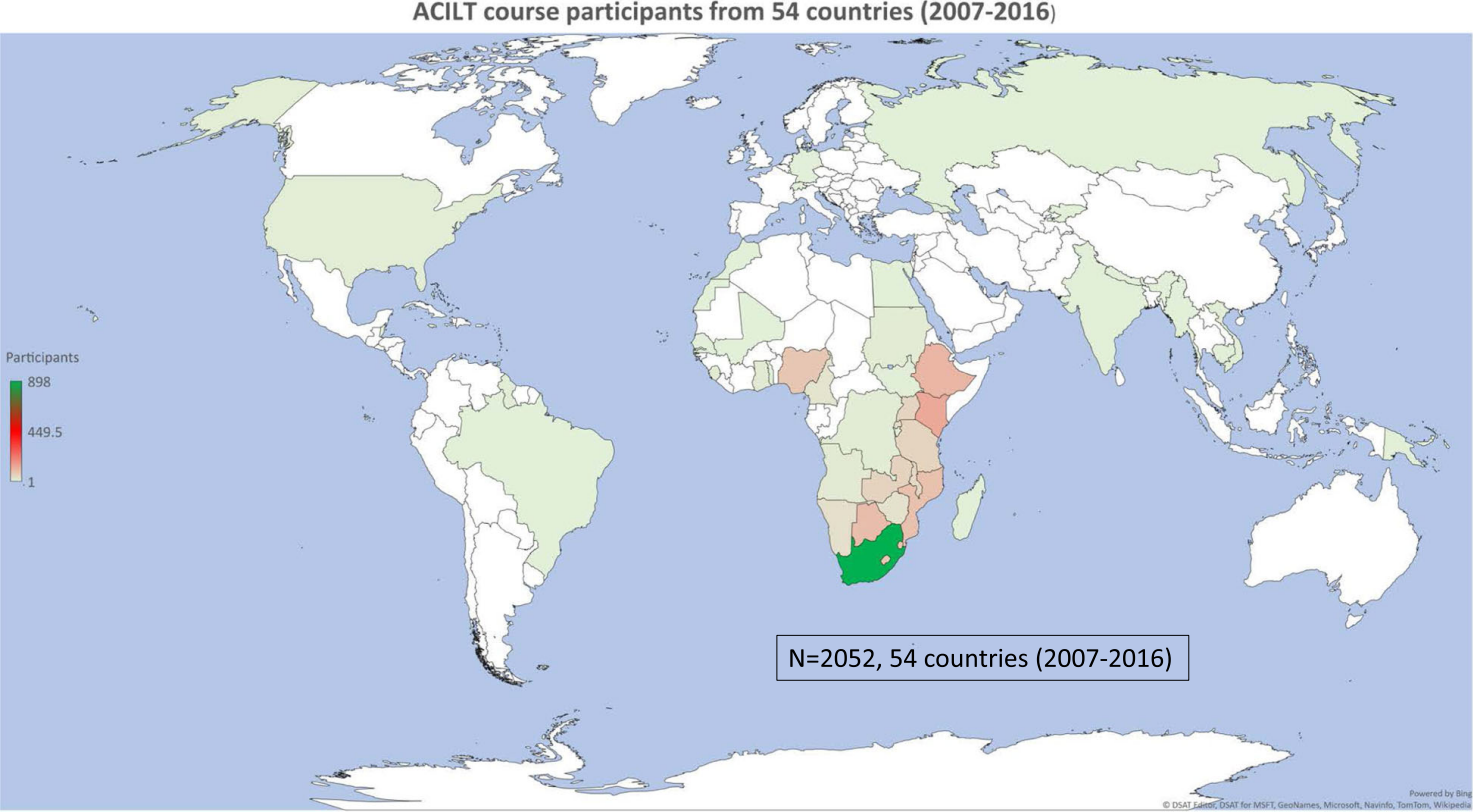
ACILT course participants from 54 countries (2007-2016). Microsoft Excel for Office 365 MSO (16.0.12527.21378) 64-bit (Centres for Disease Control and Prevention license) was used to prepare the map

Figure 1 shows the overall global distribution of countries that participated in the training and numbers of participants. South Africa had the highest number of participants (977), followed by Kenya (135) and Ethiopia (112).

### Characteristics of the participants and courses

The demographic data of the 203/867 participants who responded to the questionnaire are shown in Table 1.

**Table 1:** Demographic characteristics of responders 2008–2014 (*N* = 203)

Characteristics	Laboratory category course (***n*** = 54)	Systems strengthening category courses (***n*** = 149)
	%	%
**Country**		
Africa	86.9	78.1
Caribbean	13.0	12.5
Asia	0.0	6.3
South America	0.0	3.1
**Gender**		
Female	53.0	36.7
Male	47.0	63.3
**Age group**		
20–24	2.6	0.0
25–34	45.2	16.8
35–44	35.7	34.8
45–50 and above	16.5	48.5
**Laboratory type**		
**Government**		
Reference Lab	61.0	36.0
Hospital	20.0	19.3
Health centers	0.0	3.1
**Non-government + Faith based organizations**	6.3	14.3
*Other	12.7	27.3
**Level of education**		
Secondary, Certificate, Diploma	21.7	14.2
Bachelor of Science, College, University degree	40.1	34.2
Post College	38.2	51.6
**Role in current position**		
Non-supervisory	51.3	13.7
Supervisory	47.8	79.5
**Other	0.9	6.8
**Years of work experience at current position**		
1–5	54.3	39.8
6–12	37.1	37.9
13–22	8.6	18.0
23–51	0.0	4.3
**Continued working in the same job since the course**		
No	27.1	19.3
Yes	72.9	80.8

* Responders included laboratories supported by CDC, Donor agencies, Academic institutes, Implementing partners, Government/Public health Lab, Parastatal and Research Laboratories in “Other” category

**Responders included Technical Operations, Procurement clerk, Support staff, Senior technologist, Researcher, Quantification officer, QA and safety officer, Medical Technologist, Laboratory Technical Advisor, Laboratory Informatics Officer, Equipment Maintenance Department in “Other” current positions held

The responders were primarily from Africa, representing 173 laboratories or institutions, 61.0% of which were reference laboratories. 53.0% responders in the laboratory diagnostics category were females, while the systems strengthening category courses had more male respondents (63.3%). 79.5% responders in the systems strengthening category had supervisory experience and more than half had post college degrees. More people employed in laboratory category changed jobs (27.1%) than those employed in organizations responsible to strengthen laboratory systems (19.3%).

Table 2 summarizes the frequency of offered courses between 2008 and 2014, the number of countries who sent participants to attend these courses, and finally the response rates of the participants to the questionnaire. VL/EID was the most offered courses, (predominantly using Roche equipment) and was attended by 184 participants from 26 countries with a response rate of 15.2%. In the laboratory systems strengthening category, Biosafety and Biosecurity was the most offered course and was attended by 402 participants, from 32 countries. It had a response rate of 25.8% (103/402). Of the 198 participants trained in laboratory systems strengthening category courses, excluding Biosafety and Biosecurity, 30.3% (60/198) responded. Response rates to the questionnaires for the seven courses varied from 15.2% (VL/EID) to 48.1% (Laboratory Information System). Overall response rate was 23.4% (203/867).

**Table 2:** Summary of 867 course participants receiving survey questionnaires for 75 ACILT’s courses (2008–2014)

Course Category	Course name	Number of times course offered	Number of countries*	Number of people trained	% of Trainees responding to the questionnaires
**Laboratory Diagnostics**	HIV Viral Load Testing and Early Infant Diagnosis	35	26	184	15.2%
	HIV Drug Resistance	6	17	53	24.5%
	HIV Incidence Assay	1	11	30	43.3%
	**Sub Total**	**42**		**267**	
**Laboratory Systems Strengthening**	Biosafety and Biosecurity	20	32	402	25.8%
	National Laboratory Strategic Planning	6	27	79	26.6%
	Supply Chain Management Systems	5	17	92	28.2%
	Laboratory Information Systems	2	11	27	48.1%
	**Sub Total**	**33**		**600**	
	**Total**	**75**	**867**		

*Note: Countries sent participants to multiple courses

### Outcomes of quantitative analyses

- **Laboratory diagnostics category**

**Fig. 2:**
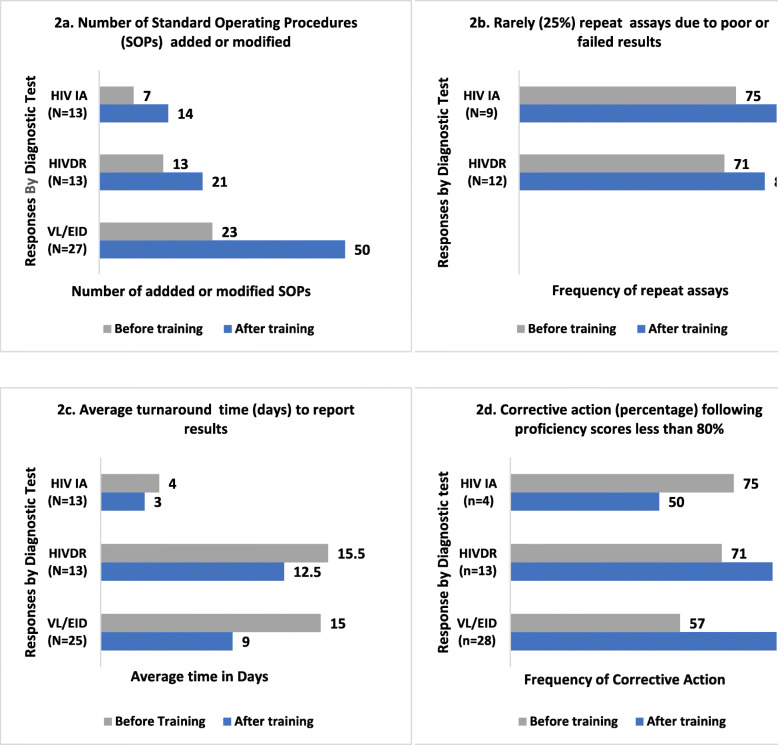
Improvements in CQI indicators for 450,000 specimens tested in 20 countries following ACILT’s training. **a**: Average number of standard operating procedures added or modified in participant’s laboratory six months before and after attending courses at ACILT. **b**: Repeat testing assays at least 25% of the time (rarely) due to poor or failed results, six months before and after attending courses at ACILT. Responders who rarely repeated genotyping assay due to poor/failed PCR results and sequence quality. This question was not administered for VL/EID courses. **c**: Reported average Turnaround time (TAT) to deliver results in days, six months before and after attending course at ACILT. TAT was measured from time of collection of specimens to return of result. **d**: Corrective action taken following proficiency testing (PT) scores less than 80%, six months before and after attending courses at ACILT. This indicator was measured in Likert scale as always (100%), usually (75%), sometimes (50%), rarely (25%), never (0%), representing frequency of corrective action for less than satisfactory (80%) PT results

Responders from 20 countries reported that six months post training, their laboratories experienced improved standardization of procedures and decreased turnaround time for a total of 450,000 specimens tested for VL/EID, HIV Drug resistance and HIV Incidence Assay (Figs. 2a-d). In addition, they had also trained an average of 502 others (data not shown). VL/EID course participants reported the most improvement. Their laboratories added and/or modified the highest numbers of Standard Operating Procedures (Fig. 2a); improved the average turnaround time from 15 to 9 days (Fig. 2c); and increased the frequency of taking corrective action for unsatisfactory proficiency scores from an average of 57 to 91% (Fig. 2d). The HIV Drug Resistance course participants reported that the average turnaround time decreased from 15.5 to 12.5 days (Fig. 2c); decreased frequency of repeat testing due to poor or failed results (Fig. 2b); increased frequency of corrective action for unsatisfactory proficiency scores from 71 to 88% (Fig. 2d).

**Fig. 3:**
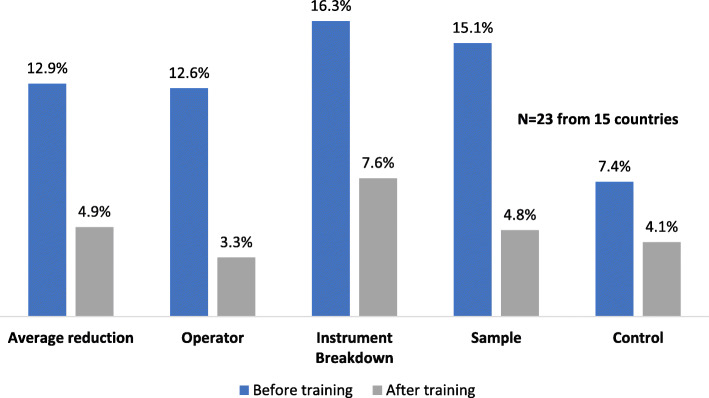
Reported reduction in error rates for 427,157 VL/EID specimens following ACILT’s courses

Figure 3 shows that in 15 countries, the overall average error rates before and after training were reduced from 12.9% to 4.9% for 427,157 tested VL/EID specimens for four key parameters: operator errors, instrument breakdown, sample errors and kit control errors.

b) **Laboratory Systems Strengthening category:**

Participants from 21 countries reported that six months after participating in the courses at ACILT, 51/60 (88.2%) made a contribution to the development of their country’s plan for National Laboratory Strategy, Supply Chain Management and Laboratory Information System (Table 3).

**Table 3:** Improvements in strengthening national laboratory systems in 21 countries six months post ACILT’s course

Course Name	Courses offered	Survey response / Total participants	Held stakeholders meeting / ** Response	Held SWOT analysis with *MoH / **Response	Produced ***SWOT document / **Response	Participated or contributed to National Plans / **Response	Access to > 25% of needed resources / ** Response	
							Before Training	After Training
National Laboratory Strategic Planning	6	21/79	11/15	16/19	14/19	16/18	2/15	3/14
Supply Chain Management	5	26/92	22/26	22/26	NA	19/20	NA	18/26
Laboratory Information	2	13/27	11/13	10/13	6/13	10/13	2/13	6/13

*Abbreviations*: *MoH Ministry of Health, ** Response Number of participants responding to the question ***SWOT Strength, Weakness, Opportunities and Threats, *NA* Question not asked, hence not applicable

**Fig. 4:**
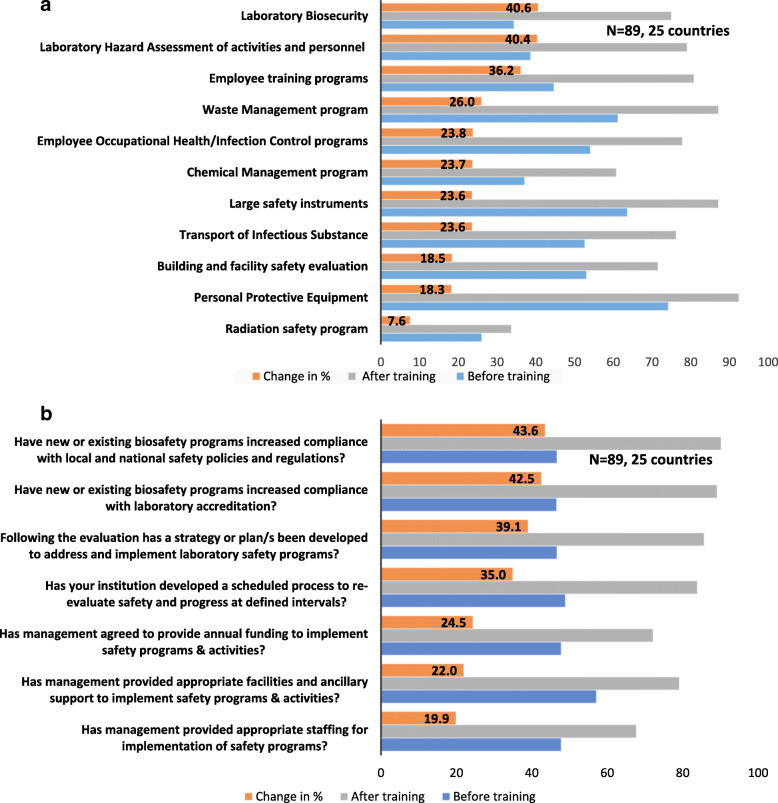
**a**: Improvements at institutional level in laboratory Biosafety programs, six months before and after ACILT’s training. **b**: Institutional gaps addressed for laboratory Biosafety program, six months before and after ACILT’s course

A majority (91%) of participants from 25 countries who participated in Biosafety and Biosecurity courses reported being involved in the development of strategies for strengthening these programs in their facilities. With the 10 elements of laboratory Biosafety and Biosecurity program evaluated, one category of large safety equipment was further subdivided into personal protective equipment (PPE) and equipment; and the results showed improvements in participant’s facilities (Fig. 4a). The highest change in percentage in safety programs were in: Laboratory Biosecurity (40.6%), Laboratory Hazard Assessment of activities and personnel (40.4%), and employees’ training programs (36.2%). Conversely, the lowest change in percentage were observed in Radiation Safety (7.6%), PPE (18.3%), Building and facility safety evaluation (18.5%), and Transport of Infectious Substances (23.6%). Figure 4b summarizes change in percentage in strategies, processes, and procedures in addressing gaps and implementing safety programs in participant’s facilities. The highest change in percentage were observed in increased compliance with local and national safety policies and regulations (43.6%), increased compliance with laboratory accreditation (42.5%) and strategies/plans developed to address and implement laboratory safety programs as a result of this evaluation (39.1%). The lowest change was reported for staffing to implement safety program (19.9%), and management providing appropriate facilities and ancillary support to implement programs and activities (22.0%).

### Factors impacting knowledge transfer in the qualitative analysis

Sixty-five of the 203 responders reported that they had trained an average of 71 people in their laboratories, organizations, countries and even neighboring countries (data not shown). Key factors affecting transfer of knowledge in the participant’s workplace is shown in Table 4. In both laboratory diagnostics and system strengthening categories, positive factors affecting knowledge transfer after the training were team and management support including MoH and implementing partners. Most reported challenges for implementing system strengthening courses were lack of funds and management support. Resource constraints and staff’s resistance to change were reported as the most obstructing factors in laboratory diagnostic course category. Key recommendations for future course considerations were resource mobilization, extended duration of courses and more refresher courses.

**Table 4:** Key factors affecting transfer of knowledge in the field, six months after ACILT’s course (2008–2014)

Name of course	Positive factors	Limiting factor to access resources	*Challenges for implementation of ACILT’s course work	*How can the course be improved?^**&**^
National Laboratory Strategic Plan	Team work and staff commitment, Support from management	Question not asked	Funding, Financial Management: Availability of pledged funds, Lack of support from management	Showcase country progress at various avenues
Laboratory Information system	Question not asked	Question not asked	Funding, Lack of support from management/government	Extend duration, Add **GIS overview
Biosafety and Biosecurity	Question not asked	Funding, Lack of support from management	Funding: Lack of budget and dependency on donor funds, Lack of support from management	Course design and structure, More practical training, Focus on additional specialized risk assessment modules, Certification and support to start the program.
Supply Chain Management system	Partner collaboration, Support from Management, Organizational management	Funding, Poor data management	Availability of data, Funding	More training and refresher courses
HIV Viral load/Early Infant Diagnosis	Support from management, Team work and staff commitment	Question not asked	Individuals resistant to change, Transport issues, Instrument errors	Extend duration and add refresher courses, Course design and more practical training
HIV Drug resistance	Question not asked	Question not asked	Staff resistant to change, Technical issues	Extend duration and change course design and structure
HIV Incidence Assay	Previous BED experience, Support from management, IT department and CDC	Question not asked	Lack of support from management	More practical training and more equipment for practical training

*Barring responses from Laboratory Information System and HIV Drug Resistance, all others are overlapping, and not mutually exclusive

^&^ Recommendations for specific topics for future courses can be made available to eligible organizations planning on similar courses

**GIS Geographical Information Systems

## Discussion

Our study shows that training offered at ACILT facilitated improvement in several technically complex areas of HIV diagnostics, importantly, in the quality and timeliness of HIV VL results in resource-limited areas. PEPFAR and other laboratory partners’ investment in a training institute providing didactic and hands-on training program in sSA was key in preparing MoH, laboratory strategic planners and policy makers, laboratory experts, supervisors and bench scientists to focus on laboratories and associated systems to keep pace with the programming needs for sustained HIV epidemic control. Importantly, the trainings reached far more individuals than those attending the courses. Unprecedented progress was made in strengthening national laboratory systems through planning and enhanced organizational efforts. It is only in recent years that laboratories in resource-limited settings have received significant support in training and instrumentation to offer these tests to their populations suffering from HIV/AIDS. Thus, providing the training to in-country laboratory staff was, and continues to be, essential to ensure the accuracy of these tests for appropriate patient care.

Although organizations invest billions of dollars in training every year, yet many competencies reportedly fail to transfer to the workplace [23]. Our results have shown that PEPFAR and other partners’ investments in ACILT have made positive impact in building country ownership to provide quality and timely laboratory services to patients in sSA countries.

Training effectiveness was reported to improve accuracy and timeliness of HIV VL testing services creating a sustainable pathway to cost savings across countries with high burdens of HIV/AIDS.

To meet the UNAIDS 90–90-90 treatment target every person starting HIV treatment will need to have access to reliable and timely VL testing and monitoring [24]. Multiple barriers prevent optimal access and uptake of VL test results including delayed and inconsistent delivery of test results to patients, errors due to equipment breakdown, unsafe biological waste management and dearth of adequate numbers of competent workforce [25, 26]. The important improvements reported in accuracy and timeliness of HIV VL testing following technical training at ACILT indicates that the participants transferred the knowledge and skills to their home laboratories, with the potential for improved patient outcomes. There is a significant association between decreased VLs, positive clinical outcomes and reduced transmission of the virus [27]. Suppressing VL helps to minimize the risk of developing resistance to the drugs taken, thus prolonging the effectiveness of therapy. All these important aspects are impacted by the quality of HIV VL testing. Also, since laboratory test errors require retesting of specimens, a decrease in the average error rate for the HIV VL/EID test would result in fewer test kits needed, and cost savings, especially for this relatively expensive test. Thus, at an average reduced error rate from 12% to 5% for the reported 427,157 VL tests, the estimated cost of repeating 29,901 fewer tests at $24.63/test is a cost savings of $736,461 for the six-month period, and the potential for savings of over $1,400,000 per year [28].

Improvements in testing accuracy, efficiency and timeliness were also reported from participants following training in HIV Drug Resistance. HIV Drug Resistance testing remains a cornerstone of ensuring effective antiretroviral therapy programs and preventing and monitoring the development and transmission of resistant HIV. Despite multilateral efforts in implementing low cost and dried blood spot-based technologies for HIV Drug Resistance testing access to this test remains a critical challenge [29]. The limited HIV Drug resistance surveillance and monitoring surveys performed so far have provided valuable data to inform treatment policies and regimens for a given country or populations [30].

HIV incidence rates are needed to measure the extent to which HIV transmission is occurring in a population, to measure the impact of interventions, to inform policy makers, and to guide HIV programming decisions [31]. A recommendation from respondents following HIV Incidence Assay training was to conduct the training on-site and on a small-scale just prior to implementation of a population study.

Biosafety and Biosecurity are critical elements of laboratory programs and often affect the quality of healthcare worker safety, staff retention, overall work environment and the patient community - also evidenced by ongoing Coronavirus 2019 (COVID-19) pandemic [32, 33]. The high attendance of this course suggests that knowledge in this area was limited. Several international partners including World Health Organization have identified gaps in Biosafety and Biosecurity programs in sSA, indicating the need for a safe work environment for patients and laboratory staff [34, 35]. These partner efforts were not captured in this evaluation. However their shared responsibility led to concurrent additional support, direct funding, expertise, and training towards these efforts [36]. The course improved participants’ knowledge and awareness in safety programs and facilitated a mitigation movement by leadership of a number of common safety concerns in laboratory and healthcare facilities across sSA. Our results indicate that programs to assess use of PPE, large safety equipment e.g., Biological Safety Cabinets, Autoclaves, Centrifuges for calibration and maintenance were ranked among the areas of least improvement. Participants reported that this area had a comparatively better performance indicators prior to ACILT’s training, hence it recorded a low improvement (Fig. 4a). Our results also indicate challenges in the areas of building and facility safety and transport of infectious substance. A growing concern that is receiving more global attention recently is the need for safe disposal of waste generated from VL/EID tests. With growing number of tests globally for COVID-19, there will also be need for trained personnel to dispose waste from COVID-19 testing responsibly. Leveraging existing human and financial resources in bio-risk management can potentially overcome some of these challenges [36]. In the most affected areas for COVID-19 access to PPE for health care workers was a pressing concern. Some medical staff were waiting for equipment while already seeing patients who may be infected or are supplied with equipment that might not meet requirements (33).

Establishing National Laboratory Strategic Plans is an essential step in securing funds and advocating for sustainable laboratory health systems in resource-poor settings [14]. Respondents to the laboratory system strengthening course questionnaires, reported that they contributed to developing the country’s first National Laboratory Strategic Plan, Supply Chain Management Systems or Laboratory Information Systems plans (Table 3). We are delighted to see that with training ACILT provided alongside in-country assistance as of January 2017, 39 countries developed or were in the process of developing their National Laboratory Strategic Plans [37]. However, lack of financial resources to advocate for National Laboratory Strategic plan for 14 respondents was also noticed. This indicates an unmet need to improve allocations at national level to fund these plans. Ethiopia was one of the countries to implement and evaluate the performance of the first five - year National Laboratory Strategic Planning and showed improvement of laboratory services after the implementation of the plan [38].

Our study reports how the trainees successfully transferred knowledge and skills gained during training to others and led to positive changes in work performance. In other studies it was noted that work environment (inclusive of transfer climate, support, opportunity to perform), exhibited the strongest, most consistent relationships with the transfer of training [23]. Because 203 responders trained an average of 71 people in their surroundings, we estimated that a minimum of 4615 have reaped the benefits of ACILT training indirectly from the evaluation of seven courses in this study. Thus, the effectiveness of training courses at ACILT are far beyond those individuals directly benefitting from the training. When considering all the courses offered by ACILT over the nine years, significantly more individuals, laboratories and laboratory systems would be benefitted than the 2052 participants trained directly at ACILT.

In addition, our findings are corroborated with subject matter experts, technical assistance visit findings to the countries and PEPFAR reports [20–22]. Thus, investments of PEPFAR and other partners in training institutions, such as ACILT are effective in building country ownership and strengthen laboratory systems to provide quality and timely diagnosis and monitoring services to control HIV epidemic and the capacity that is built can be leveraged to combat other diseases.

### Leveraging the gains from ACILT to combat HIV epidemic for COVID-19 pandemic

As of Oct 23rd 2020 the cumulative global cases for COVID-19 rose to 41,332,899 [39], with Africa contributing 1,685,589 - the lowest number of cases reported from a continent [40]. The low numbers of COVID-19 cases in Africa are a testimony to the robust laboratory capacity that ACILT built with stronger quality management systems for infectious disease diagnosis, equipment management systems, supply chain management systems, Biosafety and Biosecurity programs, accreditation readiness, improving turn-around time, laboratory strategic planning - all contributing factors to it’s preparedness and transferable capacity to upscale COVID-19 diagnosis and testing. ACILT enhanced capacities in sSA to strengthen national laboratory systems and networks, boosted competency of workforce to perform complex Molecular Diagnostic (Roche and other equipment) and serological assays that have gained emergency use approval to diagnose COVID-19 [41]. Specifically, among the strategies to upscale COVID-19 testing in African countries, a key strategy for PEPFAR and CDC Africa is to ensure leveraging existing platforms that have been the backbone for large-scale testing for HIV and tuberculosis [42, 43]. The availability of high-throughput machines, technical resources, infrastructure and the near point-of-care platforms provided through the GeneXpert machines - all capacities developed by the national HIV/AIDS and TB control programs with the support of their major partners - The United States Government and The Global Fund to Fight AIDS, Tuberculosis and Malaria has been recognized as a critical resource which can be leveraged to support the COVID-19 response [42, 43].

For example one of the many sSA countries leveraging Molecular Diagnostic testing capacity is Nigeria where the Federal Ministry of Health through the Nigeria Centre for Disease Control is leveraging about 37 molecular laboratory equipment platforms currently deployed and in use within the PEPFAR-supported National PCR Network for HIV VL/EID testing to develop COVID-19 testing capacity in every state in Nigeria [44].

### Limitations

There are limitations in our study. First, we had a low response rate (23.4%) partially due to limited access to internet in sSA countries compounded by the mass transition of email addresses by PEPFAR programs in 2015. A meta-analysis of 39 studies showed that web survey modes have on an average a 10% lower response rates than mail surveys [45]. In another self-reported web-surveys study the overall non-response rate was higher in the self-administered mode (37.9%) than in the face-to-face interview mode (23.7%) [46]. In light of these studies the response rate in this study is modest. Second, the data were retrospectively collected and self-reported, which could be subject to social desirability, personal and recall biases. Third, this is a cross-sectional impact evaluation study, which limits our capabilities to analyze the long-term outcomes of the training courses on public health system improvement and patient care service enhancements. The SLMTA training outcome and impact analysis offered at ACILT did report the long-lasting laboratory program improvements after the training [18]. Even with a modest response rate it is evident that participants were able to transfer knowledge and skills to significant number of other experts. In addition, we would like to acknowledge the contributions of other national and international organizations who are working towards the same goal.

## Conclusion

Dearth of trained and adequate laboratory workforce has hampered success of many programs for years including HIV and TB [47]. As the world moves towards electronic modes of communication and training, didactic institutes providing hands-on training for laboratory techniques remains critical for effective transference of skills and knowledge and must be continued to be funded. This laboratory training centre with a global clientele contributed to improving existing laboratory services, systems and networks, provided a sustainable pathway for cost savings for HIV services and is now being leveraged for COVID-19 testing that has infected 41,332,899 people globally.

## Supplementary Information

**Additional file 1:** HIV-1 Early Infant Diagnosis and/or Viral Load Testing Training Course - Participant Questionnaire.

**Additional file2:** HIV-1 Drug Resistance Interpretation in Sequence Editing and Data Management Processes Training Course - Participant Questionnaire.

**Additional file 3:** HIV-1 Incidence LAg-Avidity Training Course - Participant Questionnaire.

**Additional file 4:** Laboratory Information Systems - ACILT Workshop Evaluation Questionnaire.

**Additional file 5:** Supply Chain Management Systems- ACILT Program Evaluation Questionnaire.

**Additional file 6:** National Laboratory Strategic Planning and Policy – ACILT Program Evaluation Questionnaire.

**Additional file 7:** Laboratory Biosafety & Infrastructure Course - ACILT Program Evaluation Questionnaire.

## Data Availability

The datasets used and/or analyzed during the current study are available from the corresponding author on reasonable request.

## Abbreviations

ACILT: African Center for Integrated Laboratory Training
AIDS: Acquired Immune Deficiency Syndrome
CDC: Centers for Disease Control and Prevention
COVID-19: Coronavirus disease 2019
CQI: Continual Quality Improvement
HIV: Human Immunodeficiency Virus
ILB: International Laboratory Branch, CDC
MoH: Ministry of Health
PPE: Personal Protective Equipment
sSA: sub-Saharan Africa
SLMTA: Strengthening Laboratory Management Toward Accreditation
TB: Tuberculosis
USAID: United States Agency for International Development
VL/EID: Viral Load/Early Infant Diagnosis
