# Supplementary material for: Leveraging gains from African Center for Integrated Laboratory Training to combat HIV epidemic in sub-Saharan Africa
Source: BMC Health Serv Res. 2021 Jan 6;21:22. doi: 10.1186/s12913-020-06005-8 (PMC7787229; doi:10.1186/s12913-020-06005-8)
Supplement: Supplementary file 1 — Additional file 1: HIV-1 Early Infant Diagnosis and/or Viral Load Testing Training Course - Participant Questionnaire. [file 12913_2020_6005_MOESM1_ESM.pdf]

HIV-1 Early Infant Diagnosis and/or Viral Load Testing Training Course Participant Questionnaire

1. Demographics – please give CURRENT information

Name (surname, given name): ______ Age: Gender (circle one): M F

Your institution name: ______ Country name: ______

Your laboratory type (select one): O Reference O Hospital O Private O Non Government Organization

O Other, please specify: ______

Your highest education level (select one): O Primary O Secondary O Certificate

O College Degree O Post-College

O Other, please specify: ______

Your position (select one): ______ O supervisor O non-supervisor O Other, please specify: ______

Years in your position ______ Years of laboratory experience: ______ Years of HIV lab experience: ______

Are you still in the same job as when you took the course? ○ Yes ○ No ______ If no, please provide reason: ______

Has your laboratory become accredited by an external organization? ○ Yes ○ No If yes, what year: ______ Who was the accrediting body? ______

If not, provide reason: ______

2. Course-specific Information

Course Location: ______ Dates attended: ______

Course topic (select one):
O Roche COBAS AmpliPrep/COBAS TaqMan HIV-1 Qual Test for Dried Blood Spots
O Roche COBAS AmpliPrep/COBAS TaqMan HIV-1 Test for plasma viral load
O Roche COBAS AmpliPrep/COBAS TaqMan HIV-1 Qual Test for Dried Blood Spots and HIV-1 Test for plasma viral load
O Roche Amplicor HIV-1 DNA Test, V 1.5 for Dried Blood Spots
O Abbott RealTime HIV-1 Qual Test for Dried Blood Spots
O Abbott RealTime HIV-1 Test for plasma viral load
O Abbott RealTime HIV-1 Qual Test for Dried Blood Spots and HIV-1 Test for plasma viral load

Transfer of Applied Skills and Knowledge

Based on the course you took, please provide your responses in a numerical answer (such as: 0 to 9999) in the space provided or select an appropriate response to each question below. The website will not let you move to the next page till you have provided an answer to every question. If you are unable to provide an online response, a pdf format of your responses will be acceptable too.

Question	6 months BEFORE ACILT training	6 months AFTER ACILT training
Did the SOPs specific to this course exist in your laboratory?	○ Yes<br>○ No	○ Yes<br>○ No
Did you modify or add SOPs (EID and/or VL) in your laboratory?	○ Yes<br>○ No	○ Yes<br>○ No

Question	6 months BEFORE ACILT training	6 months AFTER ACILT training
List of SOPs that were modified		
List of SOPs that were added		
Did members of your laboratory review and certify the modified/added SOPs?	○ Always<br>○ Usually<br>○ Sometimes<br>○ Rarely<br>○ Never<br>○ Not yet	○ Always<br>○ Usually<br>○ Sometimes<br>○ Rarely<br>○ Never<br>○ Not yet
How often did your laboratory use or train with the modified SOPs?	○ Always<br>○ Usually<br>○ Sometimes<br>○ Rarely<br>○ Never<br>○ Not yet	○ Always<br>○ Usually<br>○ Sometimes<br>○ Rarely<br>○ Never<br>○ Not yet
How often did your laboratory use the added/revised SOPs in your lab?	○ Always<br>○ Usually<br>○ Sometimes<br>○ Rarely<br>○ Never<br>○ Not yet	○ Always<br>○ Usually<br>○ Sometimes<br>○ Rarely<br>○ Never<br>○ Not yet
How often did you successfully perform instrument troubleshooting?	○ Always<br>○ Usually<br>○ Sometimes<br>○ Rarely<br>○ Never	○ Always<br>○ Usually<br>○ Sometimes<br>○ Rarely<br>○ Never

3. Change in Results and Processes

Based on the course you had taken, please provide an answer (Yes/No, **numerical answer** e.g., 0 to 9999, pick from multiple choice). The website will not let you move to the next page till you have provided an answer to all the questions.

Question	6 months BEFORE ACILT training	6 months AFTER ACILT training
What percent of the test run failed in your laboratory due to the following errors?<br>Operator errors<br>Instrument errors<br>Sample errors<br>Control errors		
On what percent of EID Proficiency Testing panels did your laboratory score 80% or higher?		
On what percent of VL Proficiency Testing panels did your laboratory score 80% or higher?		
Did your laboratory discuss the PT panel testing results?<br>If yes, please describe the discussion outcome in less than 200 words.	○ Yes<br>○ No	○ Yes<br>○ No
Was there corrective action taken to address for the times when PT scores (EID and VL) were less than 80%?	○ Always<br>○ Usually<br>○ Sometimes<br>○ Rarely<br>○ Never<br>○ N/a	○ Always<br>○ Usually<br>○ Sometimes<br>○ Rarely<br>○ Never

Question	6 months BEFORE ACILT training	6 months AFTER ACILT training
		○ N/a
What was the total number of instrument errors in your laboratory in the last 6 months?		
What percent of instrument issues/errors corrected by you/your team as opposed to a service technician in the last 6 months?		
What was the average number of days required for your laboratory to report EID results (turn-around-time)?		
What was the average number of days required for your laboratory to report VL results (turn-around-time)?		

4. Successes and Challenges

Please answer **YES** or **NO** to each question below and provide brief comments.

Question	Answer	Comment
Have you discussed the potential to implement changes in the workplace to ensure quality testing by applying the skills you learned during the course?	○ Yes<br>○ No	
Were resources easily accessible to you for implementing the changes at your laboratory?	○ Yes<br>○ No	
Was there a person who was instrumental in providing a positive environment to implement the changes?	○ Yes<br>○ No	
Were there any other key factors that played a role in helping you to implement the changes at your laboratory? Please describe top 3. (in less than 200 words each)	○ Yes<br>○ No	
Did you encounter any challenges or barriers when implementing changes at your lab?	○ Yes<br>○ No	

5. Program-related questions (may be answered by QA manager or lab director)

How many EID specimens did you individually process on average per month in your lab? ______________________

How many VL specimens did you individually process on average per month in your lab? ______________________

How many total EID specimens did your lab process per month? ______________________

How many total VL specimens did your lab process per month ______________________

Does your laboratory have a policy in place for reporting results?<br>i) If yes,<br>a) Does your laboratory perform routine survey to assess “the customers (clinicians and/or patients) satisfaction about your laboratory service?<br>b) What percent of results reach the clinicians?<br>c) Were there any challenges or barriers experienced when reporting results to clinicians? Please describe top 3 (in less than 200 words each)<br>d) What is the perceived satisfaction rate of clinicians with the overall services of your laboratory?<br><br>ii) If no, provide reasons (in about 200 words) for not having a policy in place for reporting results and measuring customer satisfaction.	a)<br>○ Yes<br>○ No<br><br>○ Yes<br>○ No<br><br>b) 100%;75-99%;50-74%;25-49%; 1-24%; 0%<br>c) ______________________________<br>d) 100%;75-99%;50-74%;25-49%; 1-24%; 0%

Using skill and knowledge you had learnt from the course, did you train your colleagues in your laboratory?	○ Yes<br>○ No
If yes, how many colleagues in your laboratory?	
How many colleagues in other laboratories?	
If no, provide reason:	
Were there any challenges or barriers that you experienced when implementing changes to improve quality in your lab? Please describe top 3 (in less than 200 words each)	

6. Recommendations

How can this course be improved?: ____

Suggested topics or sections for future course: ____
