# Supplementary material for: Leveraging gains from African Center for Integrated Laboratory Training to combat HIV epidemic in sub-Saharan Africa
Source: BMC Health Serv Res. 2021 Jan 6;21:22. doi: 10.1186/s12913-020-06005-8 (PMC7787229; doi:10.1186/s12913-020-06005-8)
Supplement: Supplementary file 2 — Additional file2: HIV-1 Drug Resistance Interpretation in Sequence Editing and Data Management Processes Training Course - Participant Questionnaire. [file 12913_2020_6005_MOESM2_ESM.pdf]

HIV-1 Drug Resistance Interpretation in Sequence Editing and Data Management Processes Training Course Participant Questionnaire

1. Demographics – please give CURRENT information

Name (surname, given name): ______________________ Age: Gender (circle one): M F ______

Your institution name: ______________________ Country name: ______________

Your laboratory type (select one): O Reference O Hospital O Private O Non-Government Organization

O Other, please specify: ______________________

Your highest education level (select one): O Primary O Secondary O Certificate

O College Degree O Post-College

O Other, please specify: ______________________

Your position (select one): ______ O supervisor O non-supervisor O Other, please specify: ______

Years in your position ______ Years of laboratory experience: ______ Years of HIV lab experience: ______

Are you still in the same job as when you took the course? ○ Yes ○ No ______ If no, please provide reason: ______________________

Has your laboratory become accredited by an external organization? ○ Yes ○ No If yes, what year: ______ Who was the accrediting body? ______________

If not, provide reason: ______________________

2. Course specific Information

Course Location: ______________________ Dates attended: ______________________

Course name: O Practical Course on Quality Assurance of HIV-1 Drug Resistance Interpretation in Sequence Editing and Data Management Processes

3. Transfer of Applied Skills and Knowledge

Please provide your responses in a numerical answer (such as: 0 to 9999) in the space provided or select an appropriate response to each question below. The website will not let you move to the next page till you have provided an answer to every question.

Question	6 months BEFORE ACILT training	6 months AFTER ACILT training
How many SOPs for HIV Drug Resistance Genotyping did you add or modify in your laboratory for sequence analysis and interpretation? If none, skip the following questions.		
List SOPs that were added.		
How often does your laboratory use the new SOP in your lab?	○ Always<br>○ Usually<br>○ Sometimes<br>○ Rarely<br>○ Never<br>○ Not yet	○ O Always<br>○ Usually<br>○ Sometimes<br>○ Rarely<br>○ Never<br>○ Not yet

List SOPs that were modified.		
How often does your laboratory use the modified SOP in your lab?	○ Always<br>○ Usually<br>○ Sometimes<br>○ Rarely<br>○ Never<br>○ Not yet	○ O Always<br>○ Usually<br>○ Sometimes<br>○ Rarely<br>○ Never<br>○ Not yet
How were the added or modified SOPs being implemented in the laboratory? (On-the-job training, certification, etc.)		
How many colleagues have upgraded their knowledge due to your training in your laboratory on HIV-1 genotyping?		
How many colleagues have upgraded their knowledge due to your training in other laboratories on HIV-1 genotyping?		
How often do you or your team use quality sequence check measures, such as the ABI Sequence Scanner software?	○ Always<br>○ Usually<br>○ Sometimes<br>○ Rarely<br>○ Never<br>○ Not yet	○ O Always<br>○ Usually<br>○ Sometimes<br>○ Rarely<br>○ Never<br>○ Not yet
How often do you or your team use Recall, the automated HIV-1 sequence editing software? (Web or Standalone version)	○ Always<br>○ Usually<br>○ Sometimes<br>○ Rarely<br>○ Never<br>○ Not yet	○ O Always<br>○ Usually<br>○ Sometimes<br>○ Rarely<br>○ Never<br>○ Not yet
What amount of time do you spend on analyzing a run of samples from the sequencer? (Analysis includes sequence editing, contamination check, and running the appropriate drug resistance interpretation algorithm.)		

4. Change in Results and Processes

Please provide **numerical answer** (such as: 0 to 9999). The website will not let you move to the next page till you have provided an answered to all the questions.

<u>**Question**</u>	**6 months BEFORE ACILT training**	**6 months AFTER ACILT training**
How many total HIV Drug Resistance specimens did you individually process on average per month in your lab?		
How many total HIV Drug Resistance specimens did your lab process per month?		
How often does your laboratory have to repeat the genotyping assay based on poor or failed PCR results?	○ Always<br>○ Usually<br>○ Sometimes<br>○ Rarely<br>○ Never<br>○ Not yet	○ O Always<br>○ Usually<br>○ Sometimes<br>○ Rarely<br>○ Never<br>○ Not yet
How often does your laboratory have to repeat the genotyping assay based on poor sequence quality?	○ Always<br>○ Usually<br>○ Sometimes<br>○ Rarely<br>○ Never<br>○ Not yet	○ O Always<br>○ Usually<br>○ Sometimes<br>○ Rarely<br>○ Never<br>○ Not yet
How many HIV Drug Resistance Proficiency Testing Panels had scores of satisfactory or higher in your laboratory?		
Was there corrective action for the times when PT scores were less than satisfactory?	○ Yes<br>○ No<br>○ Not applicable	○ Yes<br>○ No<br>○ Not applicable
What is the average number of days required for your laboratory to report HIV Drug Resistance Genotyping results (turn-around-time)?		
Does your laboratory have a policy in place for reporting results back to the submitter?	○ Yes<br>○ No	○ Yes<br>○ No

5. Successes and Challenges

Please answer **YES** or **NO** to each question below and provide brief comments.

Question	Answer Yes/No	Comment
Have you discussed the potential to modify or add new steps in your lab to ensure the quality of testing that you have learnt in the course?	○ Yes<br>○ No	
Were resources easily accessible to you for implementing the changes at your laboratory?	○ Yes<br>○ No	
Was there a person who was instrumental in providing a positive environment to implement the changes?	○ Yes<br>○ No	
Were there any other key factors that played a role in helping you to implement the changes at your laboratory? Please describe top 3. (in less than 200 words each)	○ Yes<br>○ No	
Did you encounter any challenges or barriers when implementing quality improvement changes at your lab?	○ Yes<br>○ No	
Were there any challenges or barriers that you experienced when implementing changes to improve quality in your lab? Please describe top 3 (in less than 200 words each)	○ Yes<br>○ No	

6. Recommendations

How can this course be improved? ________________________________

Suggested topics or sections for future course: ________________________________
