# Supplementary material for: Leveraging gains from African Center for Integrated Laboratory Training to combat HIV epidemic in sub-Saharan Africa
Source: BMC Health Serv Res. 2021 Jan 6;21:22. doi: 10.1186/s12913-020-06005-8 (PMC7787229; doi:10.1186/s12913-020-06005-8)
Supplement: Supplementary file 4 — Additional file 4: Laboratory Information Systems - ACILT Workshop Evaluation Questionnaire. [file 12913_2020_6005_MOESM4_ESM.pdf]

Laboratory Information Systems

ACILT Workshop Evaluation Questionnaire

1. Demographics – please provide CURRENT information

**Name (surname):**	____________	**(given name):**	____________	**Age:**	________	**Gender (circle one):** **M F**

**Institution/ Employer:**	____	**Country Name:**	________________

**Position (select one):**	☐ Director/Manager	☐ Supervisor	☐ Non-supervisor	**Year(s) in position:**	_______________

**Name of Department/Office (for Ministry or other governmental professionals)**	____

**Laboratory type (for laboratory professional select one):**	☐ Public Health	☐ Reference	☐ Hospital	☐ Private	☐ Non-Government Organization	☐ Other, please specify:	____________

**Highest education level (select one):**	☐ Primary	☐ Secondary	☐ Certificate	☐ University/ College Degree	☐ Advanced University/College Degree	_____	☐ Other, please specify:	________ ________

**Years of ministry or government agency experience (if any):**	____

2. Course specific information

**Name of course(s):**	______________________________	**Length of course(s) in days:**	______________________
**Course Location:**	______________________________	**Dates attended:**	______________________

3. Application of Skills and Knowledge: Please provide answers to all questions

	**Questions**	**Prior to ACILT Training**		**Following ACILT Training**	
**A.**	Have you ever participated in a SWOT analysis for your Ministry of Health (identify the strengths, weaknesses, opportunities and threats) for the national Laboratory Information Systems (LIS/LIMS) strategic plan?	☐ **Yes**	☐ **No**	☐ **Yes**	☐ **No**
**B.**	Was a written SWOT analysis document produced?	☐ **Yes**	☐ **No**	☐ **Yes**	☐ **No**
**C.**	Have you ever participated in development of a national Laboratory Information System (LIS/LIMS) strategic plan?	☐ **Yes**	☐ **No**	☐ **Yes**	☐ **No**
**D.**	If yes, was a written document outlining the key components of this strategic plan produced?	☐ **Yes**	☐ **No**	☐ **Yes**	☐ **No**
**E.**	Was the national LIS/LIMS strategic plan developed prior to LIS/LIMS implementation?	☐ **Yes**	☐ **No**	☐ **Yes**	☐ **No**
**F.**	Was a multi-year LIS/LIMS strategy produced?	☐ **Yes**	☐ **No**	☐ **Yes**	☐ **No**
**G.**	Have you assisted in the development of a LIS/LIMS strategy for	☐ **Yes**	☐ **No**	☐ **Yes**	☐ **No**

	another country?		
	If yes, please list the country	**Name___________**	**Name ____________**
**F.**	Have you participated in the development of an LIS Sustainability Plan?	□ **Yes** □ **No**	□ **Yes** □ **No**
**G.**	Have you developed a Human Resources Plan to support the implementation of an LIS?	□ **Yes** □ **No**	□ **Yes** □ **No**
**H.**	Have indicators been developed to measure success of LIS implementation?	□ **Yes** □ **No**	□ **Yes** □ **No**
**I.**	Has a Request for Proposal (RFP) been developed in order to identify and contract with an LIS vendor?	□ **Yes** □ **No**	□ **Yes** □ **No**
**J.**	Has a project management plan been developed to track and manage the process of LIS implementation?	□ **Yes** □ **No**	□ **Yes** □ **No**
**K.**	List any tools that you have developed yourself or helped develop to monitor LIS success and measure indicators:	________________	___________________
**L.**	Have you been able to assess the current competencies of laboratory staff in terms of LIS implementation?	□ **Yes** □ **No**	□ **Yes** □ **No**
**M.**	Following the attendance in the course, have you been able to engage in any activities to strengthen the paper-based system?	□ **Yes** □ **No**	□ **Yes** □ **No**

4. Results and Processes:

	**Question**	**Prior to ACILT Training**	**Following ACILT Training**
**A.**	Is there currently a national LIS/LIMS strategy included in the National Laboratory Strategic Plan (NLSP?	□ **Yes** □ **No**	□ **Yes** □ **No**
**B.**	What percentage of funds in the NLSP is dedicated to supporting LIS/LIMS implementation and scale up?	0-25%<br>26-50%<br>51-75%<br>76-100%	0-25%<br>26-50%<br>51-75%<br>76-100%
**C.**	Prior to your attendance in the course, did your country have a strategic plan for LIS?	□ **Yes** □ **No**	□ **Yes** □ **No**
**D.**	Please list any partners that allocate resources for the activities and initiatives included in the national LIS strategic plan:	________________	________________
**E.**	Please list the amount or percentage of their overall lab budget allocated to LIS by these partners:	________________	________________
**F.**	Does the MOH or another area of the government have a group that provides guidance on standards for data and information systems for LIS/LIMS and other applications?	□ **Yes** □ **No**	□ **Yes** □ **No**
**G.**	Have you been a part of an LIS stakeholders' meeting for LIS strategic planning?	□ **Yes** □ **No**	□ **Yes** □ **No**
**H.**	Has an LIS team been formally created?	□ **Yes** □ **No**	□ **Yes** □ **No**
**I.**	Is a LIS Technical Working Group in place?	□ **Yes** □ **No**	□ **Yes** □ **No**
**J.**	Are there any task forces that have been developed?	□ **Yes** □ **No**	□ **Yes** □ **No**
**K.**	If yes, please state their role	________________	________________
**L.**	Have you been able to put measures in place to prevent your LIS project from failing?	□ **Yes** □ **No**	□ **Yes** □ **No**
**M.**	If yes, please list two such measures	________________	________________

**N.**	Are there any backup measures in place for the LIS?	□ **Yes** □ **No**	□ **Yes** □ **No**
**O.**	Are policies and procedures in place to be followed in the event the LIS is not functioning?	□ **Yes** □ **No**	□ **Yes** □ **No**
**P.**	If a project management plan was developed, is it used to track and manage the implementation of a LIS?	□ **Yes** □ **No**	□ **Yes** □ **No**
**Pi**	If yes, is the plan kept current with updates as needed?	□ **Yes** □ **No**	□ **Yes** □ **No**
**Q.**	If indicators were developed, have these indicators been used to measure success of an LIS during pre and post implementation?	□ **Yes** □ **No**	□ **Yes** □ **No**
**R.**	Have you developed tools to measure LIS performance, frequency of down times, frequency of problems experienced?	□ **Yes** □ **No**	□ **Yes** □ **No**
**S.**	Are there procedures and measures in place to assure data quality within the LIS?	□ **Yes** □ **No**	□ **Yes** □ **No**
**T.**	If your laboratory is working towards accreditation, has the LIS helped with this goal?	□ **Yes** □ **No**	□ **Yes** □ **No**
**U.**	Have you developed and used an LIS checklist?	□ **Yes** □ **No**	□ **Yes** □ **No**
**V.**	Can you list two types of decision making that are impacted either positively or negatively by the use of LIS?	________________	________________
**W.**	Have you been able to use the LIS to enhance overall quality in your laboratory?	□ **Yes** □ **No**	□ **Yes** □ **No**
**X.**	Have you engaged in developing an LIS budget with its various components?	□ **Yes** □ **No**	□ **Yes** □ **No**
**Y.**	Have you either been involved with hiring or assigning staff in a laboratory to manage and implement an LIS?	□ **Yes** □ **No**	□ **Yes** □ **No**
**Yi.**	If yes, were you able to base your decision on the LIS competencies?	□ **Yes** □ **No**	□ **Yes** □ **No**
**Z.**	Have you been able to define roles for staff within a laboratory to manage an LIS?	□ **Yes** □ **No**	□ **Yes** □ **No**

5. **Successes and Challenges:** Please answer Yes or No and provide brief comments

	Question	Response	Comments
**A.**	Were the skills you learned during the course used in the implementation of an LIS?		
**B.**	Did you develop or implement any initiatives for the LIS strategic planning process? Please describe briefly:		
**C.**	Describe three ways in which you were able to assist the MOH with LIS implementation:		
**D.**	What were the significant challenges that slowed or prevented the implementation of an LIS?		

6. **Recommendations**

	Question	Response
**A.**	How can this course be improved?	
**B.**	Suggested topics or sections for future courses	
**C.**	Are you still in the same job as when you took the course?	□ **Yes** □ **No**
**D.**	If no please provide the reason:	
