# Supplementary material for: Leveraging gains from African Center for Integrated Laboratory Training to combat HIV epidemic in sub-Saharan Africa
Source: BMC Health Serv Res. 2021 Jan 6;21:22. doi: 10.1186/s12913-020-06005-8 (PMC7787229; doi:10.1186/s12913-020-06005-8)
Supplement: Supplementary file 5 — Additional file 5: Supply Chain Management Systems- ACILT Program Evaluation Questionnaire. [file 12913_2020_6005_MOESM5_ESM.pdf]

Supply Chain Management Systems- ACILT Program Evaluation Questionnaire

1. Demographics – please give CURRENT information

Name (surname, given name): ______________________ Age: Gender (circle one): M F

Your institution name: ______________________ Country name: ______________________

Your laboratory type (select one): O Reference O Hospital O Private O Non-Government Organization

O Other, please specify: ______________________

Your highest education level (select one): O Primary O Secondary O Certificate

O College Degree O Post-College

O Other, please specify: ______________________

Your position (select one): ________ O supervisor O non-supervisor O Other, please specify: ________

Years in your position ________ Years of laboratory experience: ________ Years of HIV lab experience: ________

Are you still in the same job as when you took the course? ○ Yes ○ No ________ If no, please provide reason: ______________________

Has your laboratory become accredited by an external organization? ○ Yes ○ No If yes, what year: ________ Who was the accrediting body? ______________________

If not, provide reason: ______________________

2. Transfer of Applied Skills and Knowledge:

	Question	Answer	
1.	Following the course did you hold a debriefing meeting for stakeholders to share course concepts and lessons learned? And if yes, whom did you brief?	Yes	No
	MOH leadership	Yes	No
	Laboratory Directorate	Yes	No
	USG HQ (CDC Atlanta, USAID Washington)	Yes	No
	Mission leadership (PEPFAR – CDC/USAID/DoD)	Yes	No
	Other(s) (specify)___________________	Yes	No
2	Did you support the MOH lab TWG or other TWG group to conduct a sensitization and awareness meeting for national laboratory commodities strategic plan?<br><br>If not the MOH,<br>specify:____	Yes	No
3	Has there been a SWOT analysis (identify the strengths, weaknesses, opportunities and threats) for the supply chain management system in your country?	Yes	No
	Did you support the MOH to conduct a SWOT analysis (identify the strengths, weaknesses, opportunities and threats) for the supply chain management system in your country? Was this conducted before or after attending the course?	Before<br><br>Yes/No	After<br><br>Yes/No

4.	Does your country currently have a national laboratory strategic plan?				Yes	No
	If yes, which of the following areas are included in the current national plan? Please specify whether the follow areas were present before or after attending the SCMS course.<br><br>Please rate your level of contribution to the specific technical areas as appropriate (5 rated as highest) as part of the planning process.<br><br>Please also indicate whether there are plans to include these components in the future if they are currently not included.					
	**Technical Area**	**Present Before ACILT Course**	**Present After ACILT Course**	**Level of Contribution**	**Future Inclusion**	
	Harmonization	Yes/No	Yes/No	1 2 3 4 5	Yes	No
	Supply chain/logistics	Yes/No	Yes/No	1 2 3 4 5	Yes	No
	Maintenance	Yes/No	Yes/No	1 2 3 4 5	Yes	No
	Optimized procurement	Yes/No	Yes/No	1 2 3 4 5	Yes	No
	Quantification	Yes/No	Yes/No	1 2 3 4 5	Yes	No

5.	Have you supported (directly/indirectly) the development of data mechanisms to assist in data collection as evidence for improving the supply chain management system in your country? And, if yes, was this support provided before or after attending the SCMS course.			Before ACILT Course<br><br>Yes/No	After ACILT Course<br><br>Yes/No
	What data types do you routinely collect? And were they routinely collected before and/or after attending the SCMS course? Please also rate your perception of the quality of each data set mentioned.				
				**Before (1 poor/5 highest)**	**After (1 poor/5 highest)**
	Service statistics (testing uptake)	Yes	No	1 2 3 4 5	1 2 3 4 5
	Logistics data (consumption)	Yes	No	1 2 3 4 5	1 2 3 4 5
	Demographic/morbidity/targets	Yes	No	1 2 3 4 5	1 2 3 4 5
	Stock outs	Yes	No	1 2 3 4 5	1 2 3 4 5
	Instrument failures	Yes	No	1 2 3 4 5	1 2 3 4 5

3. Change in Results and Processes

Please select your response to the questions below, or provide a **numerical answer (1 to 5)** in the space provided.

	**Question**				
6.	Do you use optimized procurement procedures to inform laboratory Instrument procurement (data driven and evidence based)? If yes, was this used before or after attending the SCMS course?	Yes	No	Before	After
	Specify frequency of evidence based procurement : 1) Never; 2) sometimes; 3) mostly; 4) always	1	2	3	4
7.	Has the "12 Questions Approach" been institutionalized to make evidence based decisions regarding the procurement of laboratory equipment within your country?	Yes		No	

	If not, why ____				
8.	Do you use, or have you initiated the use of a centralized Asset Management System (AMS)? If yes, specify before or after attending the course. Also specify which core components of AMS have been implemented.	Yes	No	Before	After
	Equipment maintenance and replacement strategy	Yes	No	Before	After
	Vendor management	Yes	No	Before	After
	Contract management?	Yes	No	Before	After
	If no, please specify what your specific challenges are, and/or are there plans to do so. Clarify:				
9.	In your most recent annual laboratory quantification exercise, was a multi-method approach used? Please specify data types used, and whether used before or after attending the SCMS course. Please also indicate frequency of use.	Yes		No	
	Demographic/morbidity	Yes	No	Before	After
	How often are demographic/morbidity data used as part of national laboratory forecasting exercises : 1) Never; 2) sometime; 3) mostly; 4) always	1	2	3	4
	Service statistics	Yes	No	Before	After
	How often are service statistics (test numbers) used as part of national laboratory forecasting exercises : 1) Never; 2) sometime; 3) mostly; 4) always	1	2	3	4
	Logistics (consumption)	Yes	No	Before	After
	How often are logistics data used as part of national laboratory forecasting exercises : 1) Never; 2) sometime; 3) mostly; 4) always	1	2	3	4
	If not used, specify what data type is not used, and briefly describe why.				

4. Successes and Challenges

Please answer **YES** or **NO** to each question below and provide brief comments.

	Question	Your Answer		Comment	
10	How motivated were you to apply the skills you learned during the course to implement changes in your country?			Rate yourself: 1-5 (5 being highest)<br>1 2 3 4 5	
11	Did you conduct any innovative projects to improve the national lab commodities strategic plan/national lab instruments policy/national level instrument maintenance policy? Please describe any innovations in less than 200 words.	Yes	No		
12	How accessible were resources to you for implementing the changes in your country?<br><br>Please specify if any limitation:___________________________________			Rate: 1-5 (5 being 100% available)<br>1 2 3 4 5	
13	Was there a person who was most instrumental in providing a positive workplace environment to transfer/implement the learning from the SCMS course (specify)?	Yes	No	MOH Director	
				Organizational Management	
				Donor	
				IP	
				Other(s)<br>_________________________	

14	Please describe your top three challenges during the development of national lab commodities strategic plan in your country (keep very brief).	1.<br>2.<br>3.

5. Recommendations

How can this course be improved? ____________________

Suggested topics or sections for future course: ____________________
