# Supplementary material for: Leveraging gains from African Center for Integrated Laboratory Training to combat HIV epidemic in sub-Saharan Africa
Source: BMC Health Serv Res. 2021 Jan 6;21:22. doi: 10.1186/s12913-020-06005-8 (PMC7787229; doi:10.1186/s12913-020-06005-8)
Supplement: Supplementary file 6 — Additional file 6: National Laboratory Strategic Planning and Policy – ACILT Program Evaluation Questionnaire. [file 12913_2020_6005_MOESM6_ESM.pdf]

National Laboratory Strategic Planning and Policy – ACILT Program Evaluation Questionnaire

1. Demographics – please give CURRENT information

Name (surname, given name): ______________________ Age: ______ Gender (circle one): M F

Your institution name: ______________________ Country name: ______________________

Your laboratory type (select one): O Reference O Hospital O Private O Non-Government Organization O Public Health

O Other, please specify: ______________________

Your highest education level (select one): O Primary O Secondary O Certificate

O College Degree O Post-College

O Other, please specify: ______________________

Your position (select one): O director/manager O supervisor O non-supervisor

O Other, please specify: ______________________

Years in your position ______ Years of laboratory experience (supervisory): ______ Years of laboratory experience (non-supervisory): ______

Years of ministry or government agency experience: ______ Years of HIV lab experience: ______

Are you still in the same job with the same responsibilities as when you took the course? ○ Yes ○ No If no, please provide reason: ______________________

Has your laboratory become accredited by an external organization? ○ Yes ○ No If yes, what year: ______ Who was the accrediting body? ______

If not, provide reason: ______________________

Name of your department/office (for Ministry or other governmental professionals) ______________________

2. Course-specific information

Course Location: ______________________ Dates attended: ______________________

Course name ______________________

3. Application of Applied Skills and Knowledge:

Based on the course you had taken, please provide your responses (yes/no or a numerical answer such as: 0 to 9999) in the space provided or select an appropriate response to each question below. The website will not let you move to the next page till you have answered every question.

	Question	Before ACILT training	Following ACILT training
1	Have you ever participated in a SWOT analysis for your Ministry of Health (identify the strengths, weaknesses, opportunities and threats) to develop the National Lab Strategic Plan?<br><br>Was a written SWOT analysis document produced?	Yes/No<br><br>Yes/No	Yes/No<br><br>Yes/No
2	Have you ever participated in the development of a National Laboratory Strategic Plan for your MOH?<br><br>Was a written document outlining the fundamental areas of the NLSP produced?	Yes/No<br><br>Yes/No	Yes/No<br><br>Yes/No
3	Does the MOH or Government have a regulatory body for laboratories?<br><br>Are there plans to establish a regulatory body?<br><br>If yes, by when	Yes/No<br><br>Yes/No<br><br>Year_______	Yes/No<br><br>Yes/No<br><br>Year_____
4	Have you participated in development of a NLSP for another country?<br><br>If yes, how many? Name of country<br><br>Was a multi-year National Laboratory Strategic plan produced?	Yes/No<br><br>Number__<br><br>Name____<br><br>Yes/No	Yes/No<br><br>Number__<br><br>Name____<br><br>Yes/No

4. Results and Processes

Based on the course you had taken, please provide your responses (Yes/No; or a numerical answer such as: 0 to 9999; or select an appropriate response from the multiple choices) to each question below in the space provided. The website will not let you move to the next page till you have answered every question.

	Question	Before ACILT training	Following ACILT training
1	Is there a current National Lab Strategic Plan (NLSP) and is it aligned with the National Health Plan?	Yes/No/NA	Yes/No/NA
2	What percentage (or amount) of funds in the National Health Plan is dedicated to the laboratory network of your country?	A) 0-25%<br>B) 26-50%<br>C) 51-75%<br>D) 76-100%	A) 0-25%<br>B) 26-50%<br>C) 51-75%<br>D) 76-100%
3a<br><br>3b	How many partners contribute to the activities and initiatives included in the national laboratory strategic plan?<br>What is the percentage (or amount) of contribution by partners?	___________<br>USG<br>MOH<br>Other<br>Unknown	____________<br>USG<br>MOH<br>Other<br>Unknown
4<br><br>4b	Prior to your attendance in the course did your country have a national laboratory policy? If yes, what year?<br><br>Prior to the course did your country have a national laboratory strategic plan? If yes provide a calendar year	Yes, what year?<br>/No<br>/Don't Know<br><br>Yes, what year?<br>/No<br>/Don't Know	

5a<br><br>5b	Did you support MOH in organizing stakeholders' meetings for national laboratory strategic planning?<br><br>How many people and stakeholder organizations attended the meeting?	Yes/No<br><br>Number of people:__<br>Number of organizations:__	Yes/No<br><br>Number of people:__<br>Number of organizations:_
6a<br><br>6b	Are the roles of stakeholders described in the NLSP?<br><br>How many stakeholders share their annual work plans with MOH?	Yes/No<br><br>____________	Yes/No<br><br>____________
7a<br><br>7b	Does the NLSP include an initiative to mobilize and coordinate resources for implementation of NLSP activities?<br><br>Was a document produced to streamline the funds from partners for implementation of the NLSP?	Yes/No<br><br>Yes/No	Yes/No<br><br>Yes/No
8a<br><br>8b	Did you participate in reviewing the draft of the 5 year MOH National Laboratory Strategic Plan?<br><br>Was a final draft of National Laboratory Strategic Plan completed and submitted for MOH approval?	Yes/No<br><br>Yes/No	Yes/No<br><br>Yes/No
9a<br><br>9b	Did you participate in the development of an operational (implementation) plan based on the NLSP?<br><br>Was a plan defining 1-2 year operational initiatives and activities produced?	Yes/No<br><br>Yes/No	Yes/No<br><br>Yes/No
10b<br><br>10a	Did you participate in development of an estimated and/or designated budget for the operational plan?<br><br>Was there designated funding for the operational plan?	Yes/No/Partially<br><br>Yes/No	Yes/No/Partially<br><br>Yes/No
11a<br><br>11b	Did you support MOH in organizing the stakeholders' meeting to present concrete milestones and challenges for the operational plan?<br><br>Did partners share milestones achieved and challenges?	Yes/No<br><br>Yes/No	Yes/No<br><br>Yes/No

5. Successes and Challenges

Please answer **YES** or **NO** to each question below and provide brief comments.

	Question	Your Answer	Comment
1	Were the skills you learned during the course used in the development or implementation of strategic plans and initiatives? Please provide a brief (1 or 2 sentence) comment as an example.	○ Yes<br>○ No	

2	Did you develop or implement any initiatives or activities for the national lab strategic planning process? Please provide a brief (1 or 2 sentence) comment of each initiative or activity.	○ Yes<br>○ No	
3	Pick any 3 methods from the drop down menu in which you were able to support MOH to develop and implement the national lab strategic plan	a. Provide MOH LTWG with data<br>b. Facilitate stakeholders' discussion<br>c. Review and revise drafts of plan	
4	Did the planning process identify the personnel and financial resources for implementing the initiatives in NLSP?	○ Yes, many<br>○ Yes, some<br>○ No	
5	Were there any other factors that played a positive role to implement the changes in your country? Please describe.	○ Yes<br>○ No	
6	What were the greatest challenges that slowed or prevented development of the NLSP?	1.<br>2.<br>3.	
7	What were the challenges during implementation of the initiatives in the national lab strategic plan?	1.<br>2.<br>3.	

6. Recommendations

How can this course be improved? ________________________________

Suggested topics or sections for future course: ________________________________
