# Supplementary material for: Leveraging gains from African Center for Integrated Laboratory Training to combat HIV epidemic in sub-Saharan Africa
Source: BMC Health Serv Res. 2021 Jan 6;21:22. doi: 10.1186/s12913-020-06005-8 (PMC7787229; doi:10.1186/s12913-020-06005-8)
Supplement: Supplementary file 7 — Additional file 7: Laboratory Biosafety & Infrastructure Course - ACILT Program Evaluation Questionnaire. [file 12913_2020_6005_MOESM7_ESM.pdf]

Laboratory Biosafety & Infrastructure Course

ACILT Program Evaluation Questionnaire

1. Demographics – please give CURRENT information

Name (surname, given name): ______________________ Age: ______ Gender (circle one): M F

Your institution name: ______________________ Country name: ______________________

Your laboratory type (select one): O Reference O Hospital O Private O Non-Government Organization

O Other, please specify: ______________________

Your highest education level (select one): O Primary O Secondary O Certificate

O College Degree O Post-College

O Other, please specify: ______________________

Your position (select one): O Safety Officer O Quality Management Officer

O Lab Supervisor/Manager O Other, please specify: ______________________

Years in your position ______ Years of laboratory experience: ______ Years of HIV lab experience: ______

Are you still in the same job as when you took the course? ○ Yes ○ No If no, please provide reason: ______________________

Has your laboratory become accredited by an external organization? ○ Yes ○ No If yes, what year: ______ Who was the accrediting body? ______________________

If not, provide reason: ______________________

Course specific Information

Course Location: ______________________ Dates attended: ______________________

Course name: ______________________

II. Transfer of Applied Skills and Knowledge:

Please select your response to the questions below and provide comments in the space available.

	Question	Answer	
	**Management's Education & Responsibilities**		
1.	Does your country have national "workplace safety" regulations or laws that require employers to provide a safe workplace for employees?	Yes	No
2.	Following the course did you **hold a debriefing for stakeholders** to share course concepts, importance, and how to implement 'Laboratory Safety'? If yes, whom did you brief?	Yes	No
	Laboratory Safety Officer	Yes	No
	Laboratory Quality Management Officer	Yes	No

	Question	Answer	
3.	Laboratory Manager/ Supervisor/ Directorate	Yes	No
	Institution / Agency-Level Management	Yes	No
	Ministry Level Management	Yes	No
	Funding Partners: PEPFAR or Global Fund (please circle)	Yes	No
	Following the debriefing did the following stakeholders **acknowledge the importance** of laboratory safety and approve plan for program implementation?		
	Laboratory Safety Officer	Yes	No
	Laboratory Quality Management Officer	Yes	No
	Laboratory Manager/ Supervisor/ Directorate	Yes	No
	Institution / Agency-Level Management	Yes	No
	Ministry Level Management	Yes	No
	Funding Partners: PEPFAR or Global Fund (please circle)	Yes	No
	Other(s) (specify)___________________	Yes	No
	**Please evaluate the following results before and after training.**	**Before**	**After**
4.	Does the institution have '**policies and guidance**' which indicates that management supports the implementation of laboratory safety programs?<br><br>If no afterwards, then specify why :<br>____	Yes/No	Yes/No
5.	Has the institution provided **'resources' (workplace, funding, staff, & materials)**, to indicate that management supports the implementation of laboratory safety programs?<br><br>If no afterwards, then specify why :<br>____	Yes/No	Yes/No
6.	Are laboratory safety **strategies, goals, and objectives** being developed and implemented?<br><br>If no afterwards, then specify why :<br>____	Yes/No	Yes/No
7.	Does the institution/agency have a laboratory 'Safety Committee,' or Biosafety Committee?<br>If no afterwards, then specify why:<br>____	Yes/No	Yes/No
8.	Do the following individuals participate in your institution's safety or biosafety committee?		
	• Institution senior managing office	Yes/No	Yes/No
	• Biosafety Officer / Safety Officer	Yes/No	Yes/No
	• Laboratory Scientist (i.e., Principle Investigator)	Yes/No	Yes/No
	• Medical Personnel (Employee Occupational Health / Infection Control Provider)	Yes/No	Yes/No

Question		Answer	
	• Facility Manager/Engineer	Yes/No	Yes/No
	• Security Officer	Yes/No	Yes/No
	• Veterinary Officer / Animal Resource Manager (as appropriate)	Yes/No	Yes/No
**9. Program Evaluation:**<br>Have the following **<u>laboratory safety programs been 'evaluated & documented'</u>** to identify gaps and potential hazards/risk to the employee, institution, and the environment?			
	**A. Laboratory '<u>Hazard Assessment</u>"** of activities and personnel involved in working with biological agents, also referred to as a **'<u>Biosafety Risk Assessment</u>'?**<br>• If no, then pick one: Lack of expertise; Funding; Time constraints; Not a management priority; Other:____	Yes/No	Yes/No
	**B.** As a result of the assessment, have new or existing programs been implemented and/or strengthened?<br>• If no, then pick one: Lack of expertise; Funding; Time constraints; Not a management priority; Other:____	Yes/No	Yes/No
	**C. Employee Occupational Health / Infection Control programs**?<br>• If no, then pick one: Lack of expertise; Funding; Time constraints; Not a management priority; Other:____	Yes/No	Yes/No
	**D.** As a result of the assessment, have new or existing programs been implemented and/or strengthened?<br>• If no, then pick one: Lack of expertise; Funding; Time constraints; Not a management priority; Other:____	Yes/No	Yes/No
	**<u>E. Safety Equipment, Calibration, & Maintenance programs</u>:**<br><br>**E.1.** Assessment of **<u>Personal Protective Equipment (PPE)</u>:**<br>**E.2.** As a result of the assessment, have new or existing programs been implemented and/or strengthened**?**<br>• If no, then pick one: Lack of expertise; Funding; Time constraints; Not a management priority; Other:____	Yes/No	Yes/No
	**E.3.** Assessment of **<u>Large safety instrument:</u>** (Ex: Biological safety Cabinets, Autoclaves, Centrifuges, etc.)?<br>**E.4.** As a result of the assessment, have new or existing programs been implemented and/or strengthened?<br>• If no, then pick one: Lack of expertise; Funding; Time constraints; Not a management priority; Other:____	Yes/No	Yes/No
	**F. Building & Facility safety evaluation?** (Ex. Mechanical Sys, Ventilation Sys, Electrical<br>• If no, then pick one: Lack of expertise; Funding; Time constraints; Not a management priority; Other: ____	Yes/No	Yes/No
	**G.** As a result of the assessment, have new or existing programs been implemented and/or strengthened?<br>• If no, then pick one: Lack of expertise; Funding; Time constraints; Not a management priority; Other: ____	Yes/No	Yes/No
	**H. Building & Facility safety evaluation?** (Ex. Mechanical Sys, Ventilation Sys, Electrical)	Yes/No	Yes/No

	Question	Answer	
	• If no, then pick one: Lack of expertise; Funding; Time constraints; Not a management priority; Other: ______________________________<br><br>**I.** As a result of the assessment, have new or existing programs been implemented and/or strengthened?<br>• If no, then pick one: Lack of expertise; Funding; Time constraints; Not a management priority; Other: ______________________________	Yes/No	Yes/No
	**J. Chemical Management program evaluation?**<br>• If no, then pick one: Lack of expertise; Funding; Time constraints; Not a management priority; Other: ______________________________<br><br>**K.** As a result of the assessment, have new or existing programs been implemented and/or strengthened?<br>• If no, then pick one: Lack of expertise; Funding; Time constraints; Not a management priority; Other: ______________________________	Yes/No<br><br>Yes/No	Yes/No<br><br>Yes/No
	**L. Waste Management program evaluation?**<br>• If no, then pick one: Lack of expertise; Funding; Time constraints; Not a management priority; Other: ______________________________<br><br>**M.** As a result of the assessment, have new or existing programs been implemented and/or strengthened?<br>• If no, then pick one: Lack of expertise; Funding; Time constraints; Not a management priority; Other: ______________________________	Yes/No<br><br>Yes/No	Yes/No<br><br>Yes/No
	**N. Principles of laboratory 'biosecurity' evaluation?**<br>• If no, then pick one: Lack of expertise; Funding; Time constraints; Not a management priority; Other: ______________________________<br><br>**O.** As a result of the assessment, have new or existing programs been implemented and/or strengthened?<br>• If no, then pick one: Lack of expertise; Funding; Time constraints; Not a management priority; Other: ______________________________	Yes/No<br><br>Yes/No	Yes/No<br><br>Yes/No
	**P. Transport of Infectious Substances evaluation?**<br>• If no, then pick one: Lack of expertise; Funding; Time constraints; Not a management priority; Other<br><br>**Q.** As a result of the assessment, have new or existing programs been implemented and/or strengthened?<br>• If no, then pick one: Lack of expertise; Funding; Time constraints; Not a management priority; Other :______________________________	Yes/No<br><br>Yes/No	Yes/No<br><br>Yes/No
	**R. Radiation safety programs evaluation?**<br>• If no, then pick one: Lack of expertise; Funding; Time constraints; Not a management priority; Other: ______________________________<br><br>**S.** As a result of the assessment, have new or existing programs been implemented and/or strengthened?<br>• If no, then pick one: Lack of expertise; Funding; Time constraints; Not a management	Yes/No<br><br>Yes/No	Yes/No<br><br>Yes/No

	Question	Answer	
	priority; Other: ____		
	**T. Employee training programs review?**<br>• If no, then pick one: Lack of expertise; Funding; Time constraints; Not a management priority; Other: ____<br><br>**U.** As a result of the assessment, have new or existing programs been implemented and/or strengthened?<br>• If no, then pick one: Lack of expertise; Funding; Time constraints; Not a management priority; Other: ____	Yes/No<br><br>Yes/No	Yes/No<br><br>Yes/No
10	Does each laboratory have a 'Safety Manuals and SOPs' that are readily assessable to employees?	Yes/No	Yes/No

III. Change in Results and Processes

Please select your response to the questions below and provide comments in the space provided.

	Question		
11	Has a **strategy or plan/s** been developed to address and implement the above laboratory safety programs, as a result of the above evaluations?<br><br>If no, indicate which safety programs have not been addressed and why: ____ ____	Yes/No	Yes/No
12	Has management agreed to an **incremental strategy/plan** to address longer term improvements?	Yes/No	Yes/No
13	Has management provided **appropriate staffing** for implementation the above safety program?<br><br>If no, indicate which safety programs have not been addressed and why: ____ ____	Yes/No	Yes/No
14	Has management agreed to provide **annual funding** to implement the above safety programs & activities?<br><br>If no, indicate which safety programs have not been addressed and why: ____ ____	Yes/No	Yes/No
15	Has management provided appropriate **facilities and ancillary support** to implement the above safety programs & activities?<br><br>If no, indicate which safety programs have not been addressed and why: ____ ____	Yes/No	Yes/No
16	Has the institution and laboratories have developed a **schedule process to re-evaluate safety and progress at defined intervals**? (Ex. Quarterly, Semi-annual, Annual meetings or assessments).<br><br>If no, indicate which safety programs have not been addressed and why: ____ ____	Yes/No	Yes/No
17	Have new or existing biosafety programs increased compliance with:		Yes/No

	Question		
	• Local and national safety policies and regulations?<br>If no, indicate which safety programs have not been addressed and why: ____	Yes/No	
	• Laboratory accreditation efforts?<br>If no, indicate which safety programs have not been addressed and why: ____<br>____	Yes/No	Yes/No

IV. Successes and Challenges

Please answer **YES** or **NO** to each question below and provide brief comments.

	Question	Your Answer		Comment	
18	How motivated were you to apply the skills you learned during the course to implement changes in your country?			Rate yourself: 1-5 (5 being highest)<br>1 2 3 4 5	
19	Did you conduct any innovative projects to improve your national or institution's laboratory safety programs?<br>Please describe any innovations in less than 200 words	Yes	No		
20	How accessible were resources to you for implementing the changes at your institution?<br>Please specify if any limitation:____			Rate yourself: 1-5 (5 being 100% accessible)<br>1 2 3 4 5	
21	Was there a person who was most instrumental in providing a positive workplace environment to transfer/implement the learning from the course (specify)?	Yes	No	MOH Director	
				Organizational Management	
				Laboratory Manager/Supervisor	
				Donor	
				Implementing Partner (IP)	
				Others<br>__________________________	
22	Please describe your top 3 challenges each during the development of national lab commodities strategic plan in your country (keep very brief)	1.<br>2.<br>3.			

V. Recommendations

How can this course be improved? ____

Suggested topics or sections for future course: ____
